# Label-Free Recognition of Drug Resistance *via* Impedimetric Screening of Breast Cancer Cells

**DOI:** 10.1371/journal.pone.0057423

**Published:** 2013-03-04

**Authors:** Bilge Eker, Robert Meissner, Arnaud Bertsch, Kapil Mehta, Philippe Renaud

**Affiliations:** 1 Laboratory of Microsystems (LMIS4), École polytechnique fédérale de Lausanne, Station 17, CH-1015 Lausanne, Switzerland; 2 Department of Experimental Therapeutics, Cancer Medicine (Biochemistry), The University of Texas MD Anderson Cancer Center, Houston, Texas, United States of America; University of California Irvine, United States of America

## Abstract

We present a novel study on label-free recognition and distinction of drug resistant breast cancer cells (MCF-7 DOX) from their parental cells (MCF-7 WT) *via* impedimetric measurements. Drug resistant cells exhibited significant differences in their dielectric properties compared to wild-type cells, exerting much higher extracellular resistance (*R_extra_*). Immunostaining revealed that MCF-7 DOX cells gained a much denser F-actin network upon acquiring drug resistance indicating that remodeling of actin cytoskeleton is probably the reason behind higher *R_extra_*, providing stronger cell architecture. Moreover, having exposed both cell types to doxorubicin, we were able to distinguish these two phenotypes based on their substantially different drug response. Interestingly, impedimetric measurements identified a concentration-dependent and reversible increase in cell stiffness in the presence of low non-lethal drug doses. Combined with a profound frequency analysis, these findings enabled distinguishing distinct cellular responses during drug exposure within four concentration ranges without using any labeling. Overall, this study highlights the possibility to differentiate drug resistant phenotypes from their parental cells and to assess their drug response by using microelectrodes, offering direct, real-time and noninvasive measurements of cell dependent parameters under drug exposure, hence providing a promising step for personalized medicine applications such as evaluation of the disease progress and optimization of the drug treatment of a patient during chemotherapy.

## Introduction

Breast cancer is the most common cancer type in women and one of the leading causes of female mortality worldwide [1]. Chemotherapy is still one of the main treatment methods in clinic, causing cell death in breast tumors treated with various anti-cancer drugs. Despite the fact that many tumors initially respond to chemotherapy, cells can gain resistance and they can adapt to survive. Drug resistance often involves the transition of cancer cells from an anti-estrogen-sensitive, non-metastatic, hormone dependent phenotype to an anti-estrogen-insensitive, metastatic and hormone independent phenotype [2]. Despite the fact that there have been a plethora of studies addressing the mechanism behind drug resistance [3]–[11], a comprehensive answer to such a complex problem still remains elusive. Hence, a pressing demand has directed researchers towards the development of rapid and simple techniques for the investigation of interactions of cancer cells with drugs [12] at different stages of the disease.

Cell-based impedance spectroscopy has attracted significant attention as a label-free and non-invasive tool to study cellular properties such as cell adhesion and cell growth [13]–[15], cell migration [16], [17], stem cell differentiation [18] and the effect of anti-cancer drugs on cancer cells [19]–[21]. Although, there have been some efforts on dielectric characterization of drug resistant cells with impedance spectroscopy [22], [23] and also with dielectrophoresis technique [24], there has been no detailed study that distinguishes cell models of acquired drug resistance without genetic manipulation from their parental cells, nor investigation of drug interaction with such cells. Still substantial work needs to be done to demonstrate a comprehensive explanation why dielectric properties of cancer cells change when they gain drug resistance and how drug resistant cells interact with drugs compared to their parental wild type cells.

In addition, most of the toxicity studies demonstrated only the temporal evolution of cellular changes at one frequency where the contribution of cells to the measurement is at its maximum (cell index). As was pointed out previously [25], [26], it is possible to distinguish cellular events by measuring the impedance spectra at distinct frequencies assuming that a single cell can be described as a shell-covered sphere [27], [28]. At low frequencies (LF), the cell membrane capacitor functions as an insulator and extracellular events can be probed with impedance. At high frequencies (HF), the cell membrane capacitor is short-circuited, and the impedance changes correlate with intracellular events. Thus, impedance measurements within a wide frequency range (from Hz to MHz) allow the differentiation between the extracellular environment and the cell interior, and their changes can be probed simultaneously.

Herein, we performed real-time monitoring of drug induced cellular changes for MCF-7 breast cancer cells using impedance spectroscopy. We were able to distinguish drug resistant breast cancer cells (MCF-7 DOX) from their parental cells (MCF-7 WT) by studying their inherent dielectric properties. The effect of doxorubicin, one of the most widely used anti-tumor antibiotics, was investigated over a wide frequency range and revealed sharp differences in the temporal evolution of cellular changes between the two phenotypes. To our knowledge, this is the first study that differentiates drug resistant breast cancer cells from their parental cells based on their dielectric properties and investigates their drug response at different stages of the disease using impedance screening at different frequencies.

## Materials and Methods

### Electrode Design and Fabrication

Interdigitated (IDEs) electrodes as shown in Figure S1 were implemented in a well-based device for impedance measurements of MCF-7 cell populations. IDEs offer highly sensitive measurement owing to having large total electrode surface area. Platinum was used for electrode material since it is biocompatible [29], and highly polarizable offering mild conditions such as minimum faradaic currents and lower risk of generation of cytotoxic compounds [30]. Following evaporation of 20 nm thick titanium adhesion layer, 200 nm thick platinum layer was evaporated and patterned on a 4-inch glass substrate using standard photolithographic lift-off technique.

### Cell Lines and Cell Culture on Microelectrodes

MCF-7 cells; wild-type (MCF-7 WT) and doxorubicin resistant (MCF-7 DOX) cells (resistant to 1 µg/ml doxorubicin) were kindly provided by Prof. Kapil Mehta (Department of Bioimmunotherapy, The University of Texas M. D. Anderson Cancer Center, Houston) [31]. MCF-7 DOX are a subclone of MCF-7 WT cells that was selected by continuous exposure to doxorubicin and they are functionally and phenotypically distinct from the parental MCF-7 cells [32]. Both cell lines were maintained at 37°C and 5% CO2/95% air in RPMI 1640 culture medium (Sigma-Aldrich, Switzerland) supplemented with 10% heat-inactivated fetal calf serum (Invitrogen, Switzerland), 2 mM glutamine and antibiotics (100 U/ml penicillin and 100 µg/ml streptomycin) (Invitrogen, Switzerland). MCF-7 DOX cells were cultured in the presence of 1 µg/ml doxorubicin (LC Laboratories, USA) in order to maintain the drug-resistance phenotype. When cells are confluent inside 75 cm^2^ cell culture flasks, cells were detached with 0.25% trypsin/EDTA (Invitrogen, Switzerland), counted with hemacytometer (Sigma-Aldrich, Switzerland), and loaded inside the wells.

**Figure 1:**
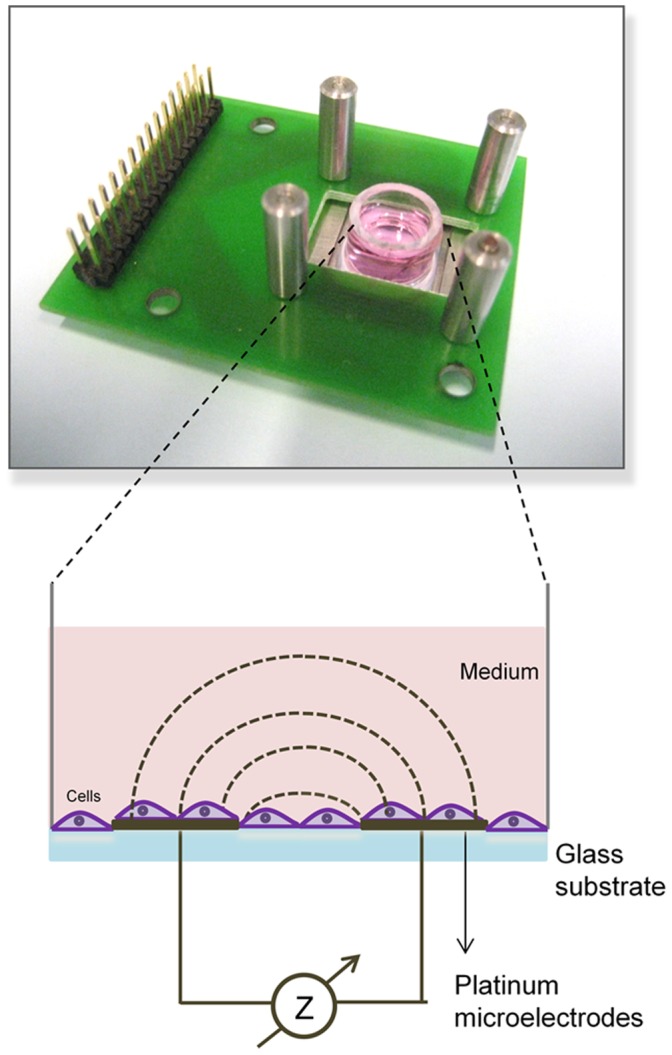
Cell culture well platform and electrical interface (top). Schematic of cell culture on microelectrodes (bottom).

Glass chips (electrodes) were autoclaved and poly(methylmethacrylate) (PMMA) wells (inner diameter = 10 mm and height = 8 mm) were disinfected with 70% ethanol before use. For each electrode chip, one PMMA well was attached on it with polydimethylsiloxane (PDMS) (Figure 1). 400 µl of cell suspension with 1 million.ml^−1^ cell density were seeded inside the well. 400000 cells on each electrode chip were sufficient enough to cover the inside part of the wells (5095 cells/mm2). The device was placed into the CO_2_ incubator and connected to an impedance analyzer (Agilent 4294A, Agilent technologies, USA) via coaxial cables, which pass through a small hole at the back of the incubator, connecting the inside of the incubator to the outside equipment.

After seeding the cells, both cell lines attached on the electrodes within 10 h as was monitored by impedance spectroscopy (Figure S2). Cells reached confluency resulting in a constant impedance signal. Potential cell proliferation in z-direction (multilayer) did not lead to further impedance increase since the current density of the electric field is not strong enough to impact the impedance signal (larger distance from electrodes). Then, doxorubicin was dissolved in cell medium and added to the wells at different concentrations (from 0.005 µM to 40 µM for MCF-7 WT and from 2 µM to 100 µM for MCF-7 DOX). The drug treatment for both cell lines was performed for 48 h. Impedance spectra were recorded from 100 Hz to 30 MHz (10 mV amplitude, 0 mV bias) using Matlab software [33]. The impedance measurements were performed at 10 mV since it corresponds to an electric field of 5 V cm^−1^ close to the electrode surface, which is three orders of magnitude lower than the membrane permeation limit of 1–4 kV cm^−1^ [34] and can be considered as safe.

### Equivalent Electrical Circuit Modeling for Cell Type Characterization

**Figure 2:**
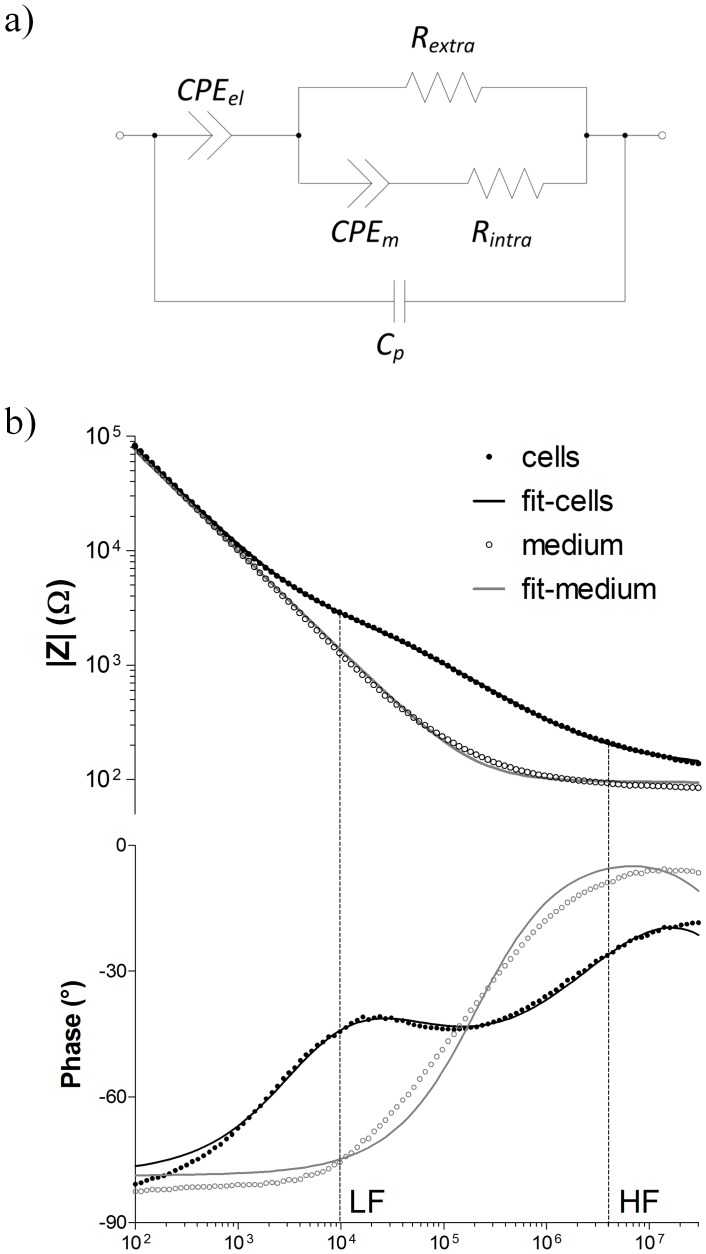
Equivalent circuit modeling. a) The cell population is represented by the Cole-Cole model with an extracellular resistance (*R_extra_*) in parallel to the tissue constant phase element (*CPE_cells_*) and a high frequency intracellular current pathway (*R_intra_*). The electrode/electrolyte interface is modeled by a constant phase element (*CPE_electrode_*). A parallel capacitance (*C_par_*) characterizes parasitic current shunts at high frequency. b) Impedance plot of MCF-7 cell culture. LF and HF refer to the low and high frequency respectively that were followed-up in this study. The equivalent circuit was used to fit the impedance and phase angle plots. (Weighted Sum of squares = 0.2247).



The electrical properties of a cell can be described with an electrical circuit, which has a membrane capacitor *C_m_* with a series intracellular resistance *R_intra_* and a parallel extracellular resistance *R_extra_* [35]. *C_m_* is replaced by a constant phase element (equation (1)) since a cell population might have variations in their properties and those of their microenvironments exerting heterogeneity within the same cell population and might result in a number of equivalent circuits with different time constants [36]–[38].
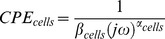
(1)where *β_cells_* is the magnitude and *α_cells_* is the exponent component of *CPE_cells_*, *ω* is the circular frequency and *j* the imaginary number. The electrode–electrolyte interface is represented by an electrode constant phase element *CPE_el_*, which describes the non-ideal capacitive behavior of the electrode due to surface roughness [39] and protein adsorption [40] on the metal surface. *C_par_* is the parasitic capacitance between the electrodes. This equivalent circuit model (Figure 2a) was shown to have a good correlation with measured impedance spectrum (Figure 2b). The weighted sum of squares (WSS) was calculated as 0.2247 based on the following equation (2).

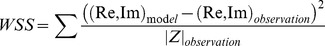
(2)Cell-dependent circuit components such as *R_extra_*, *R_intra_*, *CPE_cells_* (*α_cells_* and *β_cells_*) were obtained based on the fitting of the impedance data to the equivalent circuit model. First, the impedance spectra of the medium without cells were fitted and *CPE_el_* and *C_par_* were obtained as 5.5±0.9×10^−8^ Ω^α−1^F^α^ and (9.7±0.9)×10^−12^ F respectively. Then, the impedance spectra of both cell lines were fitted and their specific circuit components were compared.

Equivalent circuit fitting is convenient since it allows attributing values to all elements of a specific circuit model and comparing these between different cells. However, for cultures exerting low impedances (such as after drug exposure or low cell density), the fitting process becomes less reliable since more than one solution with low error becomes possible considering the number of free parameters and the less characteristic impedance curves. Therefore, we have chosen to follow-up the raw data at specific frequencies for drug effect analysis as will be described in the next section.

### Choice of Measurement Frequencies for Drug Response Studies

For drug response studies, we have recorded impedance data both at low frequency (LF) and high frequency (HF). LF is defined as the frequency before the membrane capacitor is shorted and gives information about the cell exterior. Once the membrane capacitor is short-circuited, the cell membrane is not a barrier to current anymore, the current can pass through the cell interior and information regarding the intracellular resistance can be obtained. Based on this information, the drug response studies for both cell lines were performed to extract both extra- and intracellular properties of cells.

The choice of the two frequencies is based on a whole spectrum analysis as follows. LF is ideally chosen at *f = 0 *Hz in order to avoid the impedance drop caused by the shortening of the membrane capacitor at higher frequencies. However, this is not possible at least for bipolar measurements since the impedance owing to the capacitive double layer at the electrode/electrolyte interface decreases the sensitivity for extracellular effects. Therefore, an intermediate frequency compromising both effects (interface and cell membrane capacitances) needs to be chosen. For this, we have defined LF as the frequency where the difference between the phase angle in the presence and that in the absence of cells is maximal (*φ_LF_*) (equation 3).
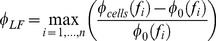
(3)where *φ_cells_(f_i_)* is the frequency-dependent impedance phase in the presence of cells and *φ_0_(f_i_) is* the one in the absence of cells. *n* is the total number of frequency points at which the impedance was measured. The impedance magnitude at this maximum phase difference frequency was found to be most sensitive to extracellular resistance changes. In our case, LF was determined as 10 kHz (Figure 2b).

HF, on the other hand, needs to be chosen high enough to avoid the impact of the membrane capacitor. HF was chosen as 2 MHz since at this frequency the phase angle was closest to 0° before being impacted by the parasitic capacitance (Figure 2b).

### Half Maximal Inhibitory Concentration (*IC_50_*) Calculation

In order to calculate the half maximal inhibitory concentrations (*IC_50_*) of MCF-7 WT and MCF-7 DOX cell lines, the normalized *|Z|_cells_* at 2 MHz were plotted *vs.* logarithm of different concentrations of doxorubicin. These inhibitory concentration-response curves were fitted with nonlinear regression by using equation (4) (GraphPad Software, USA) and *IC_50_* values were extracted.
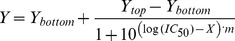
(4)where *Y_bottom_* and *Y_top_* are the values of the y-axis that correspond to the bottom and the top part of fitting curve repsectively, *IC_50_* is half maximal inhibitory concentration, and *m* is the steepness of the curve (a *m* of −1 is standard).

### Fluorescence Microscopy

For immunohistochemistry staining cells were fixed with 4% paraformaldehyde in phosphate buffer saline (PBS) (Invitrogen, Switzerland) for 20 min. After fixation, cells were treated with 3% BSA/0.1% Triton X-100 (Sigma-Aldrich, Switzerland) for 50 min. For tight junction staining, cells were first incubated with the primary antibody monoclonal rabbit-anti-occludin (1∶200, Invitrogen, Switzerland) for 2 h and subsequently with the Cy-2 coupled secondary antibody (1∶150, Dianova GmbH, Germany) for 2 h. For E-cadherin staining cells were incubated with the primary antibody monoclonal mouse-anti-E-cadherin (1∶150, Invitrogen, Switzerland) for 2 h and followed by the incubation of rhodamine coupled secondary antibody (1∶200, Dianova GmbH, Germany) for 2 h. For actin staining, cells were incubated with FITC coupled phalloidin (Sigma- Aldrich, Switzerland) for 2 h. Thereafter; all samples were incubated with 0.1 µg ml^−1^ DAPI in PBS (Sigma-Aldrich, Switzerland) for 30 min. After repeated washing, cells were observed with a confocal laser-scanning microscope (LSM 700 inverted, Zeiss, Germany).

### Time-Lapse Microscopy

Both cell lines were cultured in glass bottom culture dishes (MatTek Corporation, USA). After all the cells adhered on the glass bottom, the effect of doxorubicin at different concentrations (from 0.02 to 20 µM) on cells were monitored with time-lapse microscopy (Olympus CellR inverted, Japan) at 37°C and 5% CO_2_ during 2 days, taking measurements every 30 min. The cell morphology during drug exposure was investigated with 60× objective and phase contrast mode.

### Statistical Analysis

Dielectric components of cells such as *R_extra_* obtained for MCF-7 WT cells in 35 different chips and MCF-7 DOX cells in 20 different chips were expressed as means with 95% confidence interval. Statistical analysis of *R_extra_* was compared between MCF-7 WT and MCF-7 DOX cell populations using unpaired t-test. The observed importance level (p-value) of the difference between two means was considered significant when p<0.001.

## Results

### MCF-7 DOX and MCF-7 WT Cells Possessed Different Extracellular Properties

**Figure 3:**
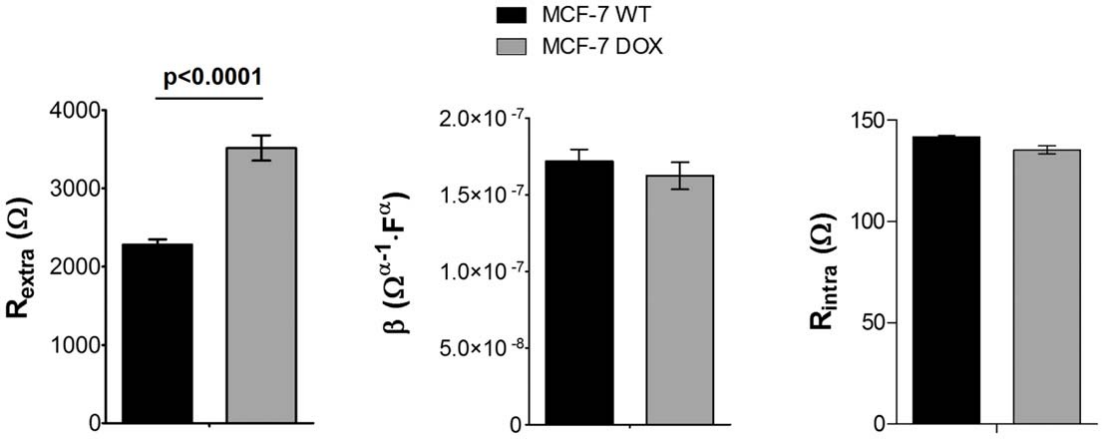
Comparison of the dielectric components of MCF-7 WT and MCF-7 DOX cells (mean ± SEM). a) The resistant cells display a significant increase in *R_extra_* compared to the wild type cells (p<0.0001). b) and c) The magnitude β of *CPE_cells_* and *R_intra_* do not show significant alterations when the cells gain drug resistance. (n = 35 for MCF-7 WT and n = 21 for MCF-7 DOX cells, cell constant = 3660±50 m^−1^ and σ_medium_ = 361 mS.cm^−1^, substrate: platinum).

We investigated whether doxorubicin resistant MCF-7 cells (MCF-7 DOX) can be singled out from their parental cells (MCF-7 WT) or not based on their dielectric properties. In order to reach this goal, the impedance spectra of MCF-7 WT and MCF-7 DOX cells with equal cell density were fitted in the absence of drug. Figure 3 shows the values of the fitted circuit components (*R_extra_*, *R_intra_*, magnitude *β* of *CPE_cells_*) for both wild type and drug resistant cells. Although *R_intra_*, and *β* showed negligible change when the cells gained resistance to doxorubicin, we observed a significant difference in *R_extra_* between these two. MCF-7 DOX cells exhibited 50% increase in *R_extra_* compared to MCF-7 WT cells when both were cultured at confluency on platinum electrodes.

**Figure 4:**
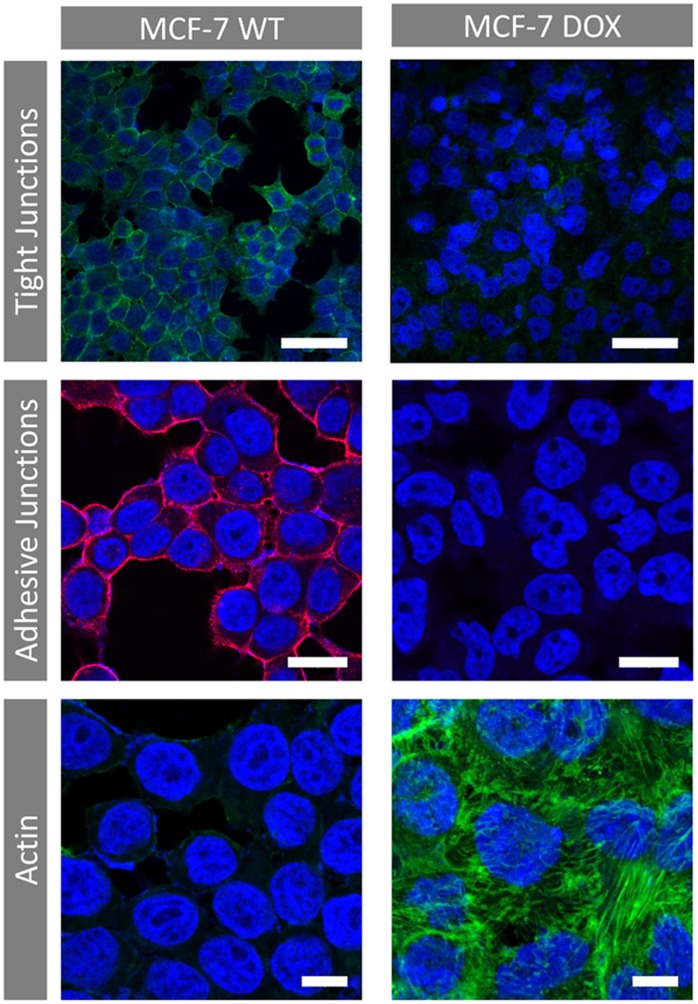
Immunocytochemical staining of cellular tight junctions (bar = 50 µm, green: occludin-Cy-2, blue: DAPI), adhesive junctions (bar = 20 µm Red: E-cadherin - Rhodamine, blue: DAPI) and actin (bar = 10 µm, green: Phalloidin-FITC, blue: DAPI) for MCF-7 WT and MCF-7 DOX cells. Loss of tight junctions and adhesive junctions is observed upon gaining drug resistance. No E-cadherin was identified for MCF-7 DOX cells. Drug resistant cells displayed a highly dense fiber-like network of actin filaments and more cell-to-cell contact compared to MCF-7 WT cells.

In order to find out the reason behind the increase of *R_extra_* when the MCF-7 WT cells gain drug resistance, we investigated the nature of MCF-7 cellular tight junctions, adhesive junctions and cytoskeleton structure by occludin staining, E-cadherin staining and actin labeling respectively (Figure 4). The presence of tight and adhesive junctions was confirmed for MCF-7 WT cells. Occludin and E-Cadherin labeling was intense and formed a continuous line at the cell contacts. No E-Cadherin could be identified for MCF-7 DOX cells and tight-junctions were very rare. Immunocytochemical staining of actin cytoskeleton revealed significant structural changes between MCF-7 WT and MCF-7 DOX cells. Drug resistant cells exhibited a highly dense fiber-like network of actin filaments while MCF-7 WT cells showed much less labeling for F-actin protein.

### MCF-7 DOX and MCF-7 WT Cells Displayed Different Impedance Response to Doxorubicin at Low and High Frequency

**Figure 5:**
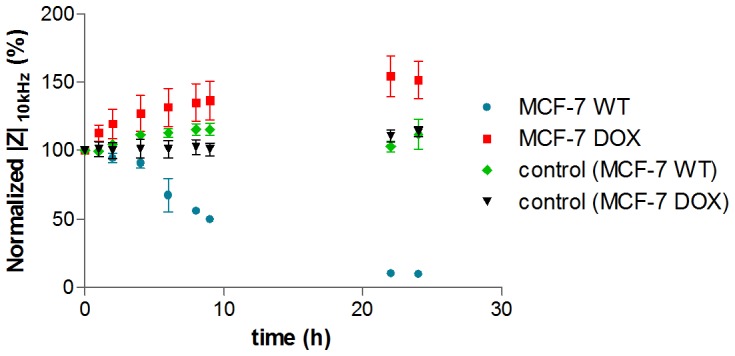
Drug response of MCF-7 WT cells and MCF-7 DOX cells to 20 µM doxorubicin during 24 h at LF (10 kHz). Data points (mean ± SEM, n = 5) were normalized to the magnitude value at t = 0 h. The impedance drops sharply at LF for MCF-7 WT cells, indicating that 20 µM doxorubicin is strong enough to induce toxic effects, while cells in the absence of drug (control) were healthy and LF signal was relatively unchanged. On the other hand, MCF-7 DOX cells displayed increasing impedance compared to their control indicating resistance to this drug concentration and demonstrating the substantial differences in the drug response of MCF-7 WT cells and their drug resistant phenotypes.

The effect of doxorubicin on MCF-7 WT and MCF-7 DOX was studied separately at LF and HF. First, MCF-7 WT and MCF-7 DOX cells were exposed to a 20 µM concentration of doxorubicin during one day of continuous exposure. Figure 5 shows time dependent impedance magnitude changes at 10 kHz. The impedance response to doxorubicin of both cell lines is substantially different. While MCF-7 WT cells were severely affected by the drug at this concentration, MCF-7 DOX cells showed increasing impedance magnitudes under drug exposure within 24 h. Control experiments showed that both MCF-7 DOX and MCF-7 WT cells were healthy in the absence of drug and displayed relatively unchanged impedance profiles within 24 h. In order to make sure the impedance magnitude changes originate from doxorubicin-induced cellular changes, not from the presence of doxorubicin in the cell medium itself, the spectra of cell medium with and without drug were recorded as a control experiment. Only a 5% increase was observed in the impedance when 20 µM doxorubicin was introduced to the cell medium (Figure S3). This is negligible compared to the observed increase of the impedance in the presence of cells. In addition, the temporal evolution of *|Z|* of 20 µM doxorubicin in cell medium also did not show any change when there are no cells inside the wells (Figure S3).

**Figure 6:**
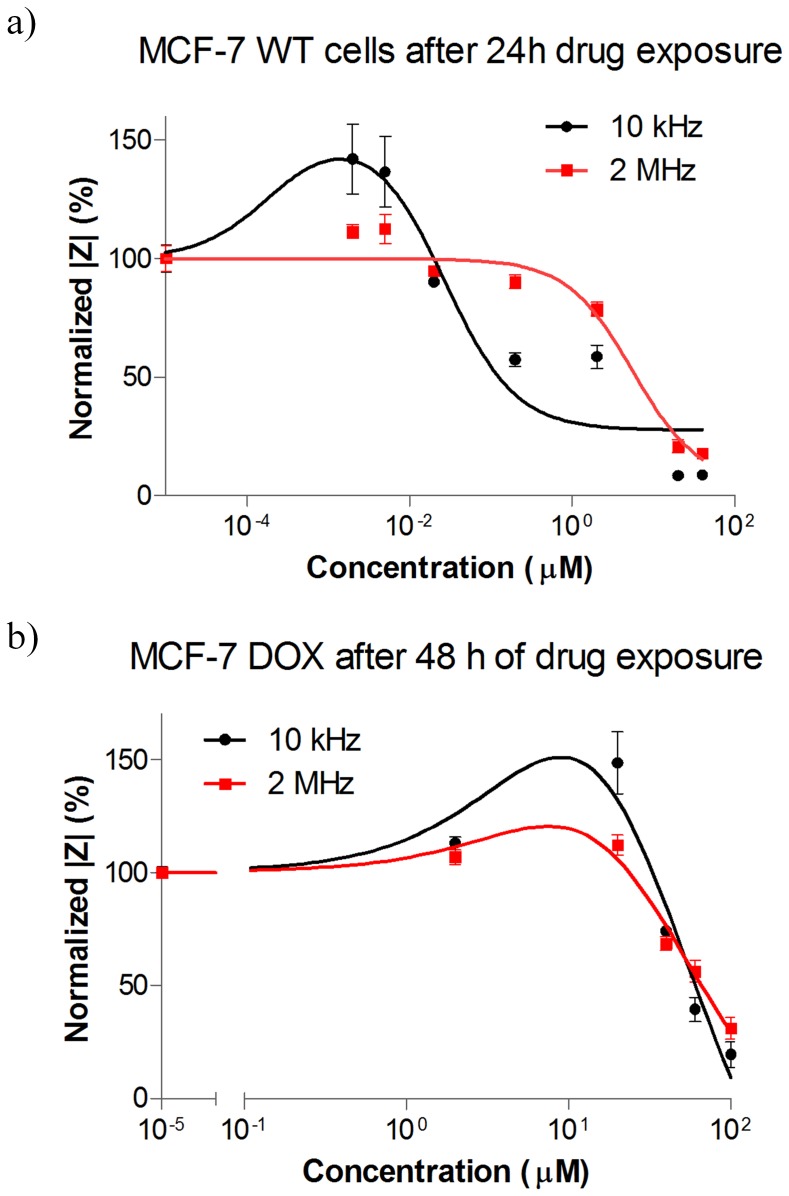
Normalized |Z| vs. different concentrations of doxorubicin at LF (10 kHz) and HF (2 MHz) for a) MCF-7 WT: the LF and HF signals exhibit different profiles with respect to different drug concentrations. At low drug concentrations, the LF signal increased when HF signal was unaltered. At mild drug concentrations such as 0.2 µM doxorubicin, the LF signal decreased with no change in HF signal. At high drug concentrations such as 20 µM and 40 µM doxorubicin both LF and HF signal decreased sharply, showing similar kinetics. b) MCF-7 DOX: the LF signal increases at non-lethal drug concentrations when HF signal exhibited a slight increase. At high drug concentrations such as 40 µM and higher, the LF and HF signal displayed similar impedance profiles and dropped sharply. The plots were fitted by nonlinear regression based on the equation for bell-shaped concentration response only for visual presentation. Data points (mean ± SEM, n = 5) were normalized to the magnitude value at t = 0 h.

Doxorubicin treatment with different concentrations was performed with MCF-7 WT cells but one-day drug exposure was enough to obtain significant cellular changes for highly sensitive MCF-7 WT cells. Doxorubicin was dissolved in cell medium and added to the cells at different concentrations (from 0.005 µM to 40 µM for MCF-7 WT and from 2 µM to 100 µM for MCF-7 DOX). As seen in Figure 6a, The LF signal exhibited an increase in the presence of low drug concentrations when HF signal was unaltered. When cells were exposed to higher drug concentrations, the LF signal reversed its direction and started decreasing after 0.02 µM drug intake, while HF signal did not change significantly up to 2 µM drug exposure and decreased much later than LF signal. Figure S4 also supports the correlation between LF and HF signals and the drug induced morphological changes of MCF-7 WT as a function of drug concentrations.

On the other hand, since MCF-7 DOX cells are highly resistant; their temporal evolution was monitored during drug exposure for a longer period of time (48 h). LF signal showed considerable increase up to 20 µM drug exposures while HF signal displayed a slight increase up to the same drug concentration (Figure 6b). Figure S5 also confirms that MCF-7 DOX cells were healthy and dense, all adhered on the platinum electrodes after 24 h of 20 µM drug exposure. High concentrations of doxorubicin (from 40 µM to 100 µM) lead to an impedance drop at both LF and HF, revealing similar kinetics and decreasing sharply at both frequencies.

**Figure 7:**
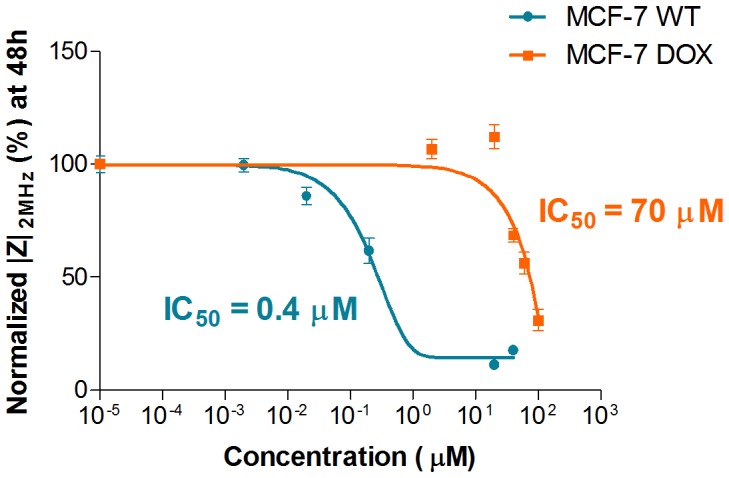
Concentration-response curves are obtained by plotting *|Z|* at 2 MHz after exposing the cells to various concentrations of doxorubicin. The data was fitted to a sigmoid model. Based on the resulting fit, *IC_50_* values were obtained as 0.4 µM and 70 µM for MCF-7 WT and MCF-7 DOX respectively. Data points (mean ± SEM, n = 5) were normalized to the magnitude value at t = 0 h.

In addition, concentration-response curves were obtained by plotting *|Z|* at 2 MHz after 48 h of drug exposure vs. different drug concentrations. The half maximal inhibitory concentrations (*IC_50_*) for both MCF-7 WT and MCF-7 DOX cells were extracted from the fit to sigmoid concentration-response curves as shown in Figure 7. *IC_50_* values were obtained as 0.4 µM and 70 µM for wild-type cells and drug resistant cells respectively.

### MCF-7 DOX and MCF-7 WT Cells Showed Remarkable LF Impedance Increase at Nontoxic Drug Doses

**Figure 8:**
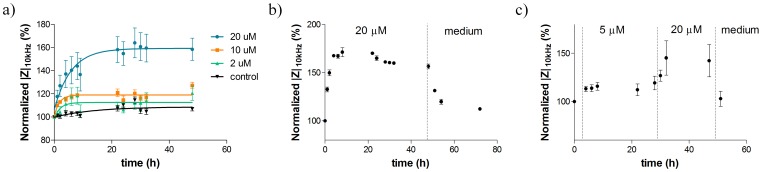
Effect of stimulatory non-toxic drug concentrations on MCF-7 DOX. a) MCF-7 DOX exhibits a concentration-dependent increase in impedance magnitudes during drug treatment with nontoxic concentrations. The plot was fitted by nonlinear regression using exponential decay equation only for visual presentation. b) Impedance magnitudes vs. time plots of MCF-7 DOX cells when 20 µM doxorubicin was applied for 48 h and followed by medium washing for 24 h. This shows that the impedance increase is reversible when the drug is removed. c) Temporal evolution of *|Z|* when resistant cells were exposed to 5 µM doxorubicin for 24 h, followed by 20 µM drug concentration for 24 h, then medium washing as a last step for 1 h. This shows that the impedance increase can be sequentially added up; once the stress on cells is removed, the initial state of extracellular environment is reobtained. Data points (mean ± SEM, n = 5) were normalized to the magnitude value at t = 0 h.

MCF-7 DOX cells showed a concentration-dependent impedance increase when they were exposed to drug concentrations between 2 µM and 20 µM and such impedance increase with respect to the control was more pronounced at LF with very little difference obtained at HF (Figure 6b and 8a). When the drug was replaced with medium after MCF-7 DOX cells were exposed to such concentrations of doxorubicin for 48 h, the impedance signals retrieved back to their initial magnitudes (Figure 8b). In addition, a step-wise increase of the drug concentration also leads to a sequential increase of *|Z|* (Figure 8c). The subsequent exposure of the resistant cells to first 5 µM and then 20 µM doxorubicin concentrations (each for 24 h) resulted in a step-wise increase of *|Z|*; finally the signal dropped back to its initial value after incubating the cells in medium for 1 h.

Such an impedance increase at nontoxic drug concentrations was also observed for MCF-7 WT cells (Figure 6a). MCF-7 WT cells displayed higher impedance when they were exposed to the nonlethal drug concentrations such as 2 and 5 nM compared to the same cells in the absence of drug (control). Such an increase was observed only at LF while HF signal showed negligible change in the presence of non-lethal drug concentrations.

## Discussion

In the present study, we have demonstrated that impedance spectroscopy is a simple and useful technique that distinguishes drug resistant cells from their parental cells based on their dielectric properties. Drug resistant variants were shown to exert higher extracellular resistance. It was found that actin cytoskeletal remodeling might play an important part in higher extracellular resistant behavior of MCF-7 DOX cells, providing stronger cellular architecture. Moreover, we have screened concentration and time dependent effects of doxorubicin on cell interior and exterior of breast cancer cells at earlier and later stages of the disease. Our experiments indicate four distinct drug concentration ranges that display particular dielectric responses of the cells under drug exposure.

### Distinction of Drug Resistant Cells with their Cell Dielectric Properties

We investigated the dielectric properties of MCF-7 DOX cells and MCF-7 WT cells. Based on fitting their impedance profiles with the equivalent circuit model, both cell lines exhibited similar *R_intra_* and *β* indicating that there were no measurable alterations in the electrical properties of the cell membrane and cytosol when MCF-7 cells gain drug resistance. However, drug resistant cells displayed 50% increase in *R_extra_*, implying significant changes in the cell morphology and structure upon drug resistance. The finding with *R_extra_* is a novel result, however quite unexpected. *R_extra_* for drug resistant cells was expected to be lower since loss of tight junctions and absence of E-cadherin in MCF-7 DOX cells (Figure 4) should have resulted in shunting of the current flow through the intercellular space and thereby lowering the *R_extra_*. On the other hand, actin staining shows that MCF-7 DOX cells possessed highly dense F-actin network and had elongated form of actin fibers in cytoplasm up to cell peripheral region, while MCF-7 WT cells had peripheral actin, which is much less dense in their cellular structure. These results suggest that remodeling of the actin cytoskeleton might probably be the reason behind the higher extracellular resistance when MCF-7 cells become resistant to doxorubicin and when seeded at confluence on platinum electrodes. In fact, in cell culture on stiff substrates, the actin cytoskeleton tends to organize in stress fibers [41]. Doxorubicin treatment disrupts such fiber-like organization [42], [43] leading to cell–substrate detachment [44].

Hence, the actin network is a crucial and complex system that provides base and support to retain cell morphology and mechanical structure [45]. The extracellular and phenotypical events of a metastatic carcinoma are mainly governed by structural reorganization of actin [46], [47]. If the MCF-7 cells use the reorganization of the actin cytoskeleton to become resistant to doxorubicin, then a question may arise about the role of dynamic actin remodeling on transformation of a cancer cell to a drug resistant phenotype. Some studies showed that actin remodeling may play a part in inactivation of some actin binding proteins such as E-cadherins [48], [49] and gelsolin [50] that have tumor-suppressor functions, and activation of actin signaling pathways that leads to malignant phenotypes such as pathways involving Ras [51] and Src [52] proteins. A recent study showed lower cell stiffness upon disrupting the actin cytoskeleton with an inhibitor of actin polymerization, implying the importance of actin remodeling in drug-mediated cell stiffness modulation and drug resistance [53]. However, it is still unclear if such highly dense F-actin network participates in drug efflux by limiting the drug intake by drug resistant cells or if such organization of F-actin filaments participates in drug efficacy through influencing the cell membrane physical properties. In addition, such actin reorganization might be a consequence of drug-induced phenotypic events of a signaling pathway that leads to drug resistance. The exact effect of actin remodeling on the drug resistance remain to be elucidated and was not our focus here, thereby was not conducted in the framework of this study.

Overall, the drug resistant cells were distinguished from their parental cells based on their higher extracellular resistance. Such detection of drug resistant cells based on their dielectric properties might allow assessing the stage of the disease by selective recognition of certain phenotypes and designing the optimum personalized treatment based on the disease progress.

### MCF-7 DOX Displayed a 175 Times Higher Drug Resistance

The drug response of MCF-7 DOX cells was compared with the one of MCF-7 WT cells. It was observed that only strong doxorubicin concentrations such as 40 µM and higher could decrease the LF and HF impedance signal of MCF-7 DOX within 48 h (Figure 6b). MCF-7 WT cells, on the other hand, already displayed an impedance decrease at much lower concentration such as 0.2 µM after 24 h (Figure 6a), showing that MCF-7 DOX cells are more resistant to doxorubicin.

Based on the concentration response curves of these two cell lines (Figure 7), MCF-7 DOX cells exhibited a 175 times higher *IC_50_* value than that of MCF-7 WT cells, which is consistent with the findings of Mehta et al. who obtained more than 150 times more resistance with MCF-7 DOX cells compared to their parental cells by applying conventional viability assay to the same cell lines [11]. This shows that impedance screening offers a highly sensitive label-free technique that can assess the effect of a drug on the different phenotypes of the same cell type with high accuracy.

### Impedance Profiles Revealed Four Distinct Drug Responses within Specific Ranges of Drug Concentrations

We already showed in our previous study that LF and HF impedance signals exhibited different kinetics when cells were exposed to drug, revealing different information about cell properties [25]. In this study, we also observed different temporal evolutions at LF and HF when MCF-7 WT cells were exposed to 0.2 µM and higher doses of doxorubicin (Figure 6a). At LF cell membranes block the current from flowing inside the cells, acting as capacitors. Such densely packed insulating cell layer exerts a high extracellular resistance and this resistance is prone to change when stress is applied on cells. The extracellular resistance drops when cell-substrate and cell-cell contacts are weaker due to the contraction of cells in the presence of drug and current flows through the intercellular space more easily. This phenomenon is the reason why the LF signal decreases upon interaction with drug [25] and renders this method much more sensitive to toxic effects compared to traditional viability assays since drug toxicity can be sensed long before cell death takes place (see Figure 4 in [10]). On the other hand, the cell membrane capacitor shortcuts at HF and cell interior contribute to the measured impedance more significantly. As we already discussed in our earlier study [25] when cells are dying, the intra- and extracellular matters exchange due to the pore formation during cell death, resulting in more conductive cytosol with the influx of more conductive cell medium [28], and thus lower HF impedance signal.

**Figure 9:**
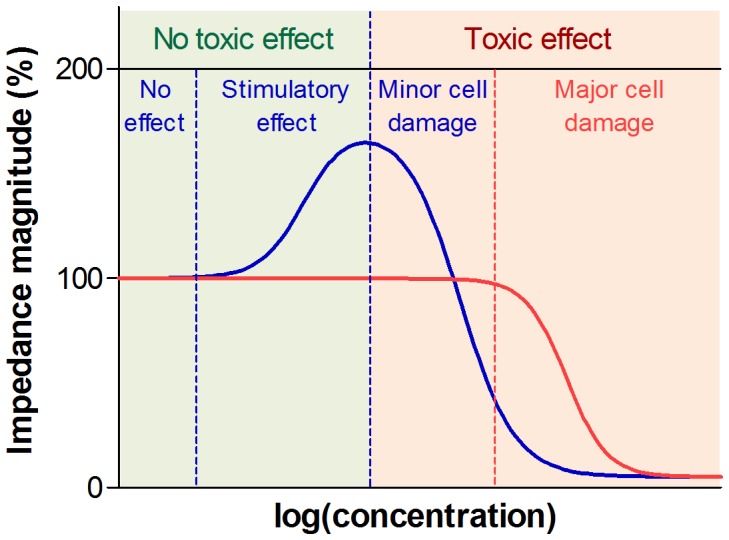
Hypothesized impedimetric concentration response map. (1) At low nontoxic drug concentrations, the drug has no effect on cells, which results in unchanged LF and HF signal. (2) There is a drug concentration range where stimulatory effect takes place and reflects in higher impedance owing to the changes in the cellular structure and cell-cell interaction. (3) At toxic drug concentrations, there are two different circumstances that need to be taken into account. (3) At mild drug concentrations, the drug induces minor morphological changes while cell interior is intact, reflecting in lower LF with HF remaining the same. (4) At high inhibitory concentrations, the LF and HF signal drop sharply due to the substantial morphological changes and major cell damage.

In addition to the difference between LF and HF profiles, we have observed two more distinct responses at LF within specific concentration ranges (Figure 9). First, the drug has no effect, resulting in unaltered LF and HF signal. Second, at higher nontoxic drug concentrations, an increase in impedance at LF is observed. The cell morphology might adapt itself in a way that cells show stimulatory response upon drug interaction and impede the current more with enhanced cell-substrate contact, resulting in higher impedance at LF while HF signal remains the same. When the drug is toxic at higher concentrations, there are two circumstances that need to be taken into account. At mild inhibitory drug doses, there might be an intermediate phase where drug induced morphological changes might result in lower LF while cell interior is unaffected with no change in HF signal. At higher toxic concentrations, drug might induce substantial changes in the extracellular and intracellular environment, reflecting in sharp and simultaneous decrease in both LF and HF signal. These findings indicate that impedance spectroscopy not only distinguishes minor morphological changes from major cellular damage based on different kinetics of LF and HF signal, but also provides useful information when applying non-toxic drug concentrations, i.e. distinguishing concentrations that induce a stimulatory response from the concentrations that result in no measurable effect.

### Impedimetric Measurements Revealed Enhanced Cell Stiffness at Non-toxic Drug Concentrations

When low concentrations of doxorubicin (2–5 nM for MCF-7 WT and 2–20 µM for MCF-7 DOX) were added to the cell medium, *|Z|* showed remarkable increase, especially at LF, compared to that of cells in the absence of drug (Figure 6). The HF signal was relatively unchanged. In addition, there was no impedance change when such drug concentrations were added to the cell medium itself in the absence of cells. These results imply that drug at low and non-toxic concentrations might influence the biophysical and biomechanical properties of cells as hypothesized in Figure 9 and the cellular structure might adapt its organization in a way to fight against the drug by gaining cell stiffness. In fact, while a disease state causes biological and functional changes in cells, it also leads to the significant changes in the morphological properties of cells [54]. Cytoskeletal actin has a crucial role in governing mechanical properties of cells [55], [56] and it is prone to dynamic changes during progression of cancer in all forms [54]. Some studies showed increased cell stiffness when normal cells evolve to be cancerous cells [57] and some showed reduced cell stiffness upon metastasis using different biomechanical assays [58], [59]. Moreover, it was revealed that chemotherapy drugs such as taxol and cisplatin resulted in the increase in cell stiffness of metastatic cancer cells [60] and increased cell stiffness of drug sensitive ovarian cancer cells upon sublethal concentrations of cisplatin was shown by using atomic force microscopy (AFM) [53]. Here, we also observed pronounced and intense changes in the actin cytoskeleton structure and organization when cells gain drug resistance. This indicates that cell stiffness might play an important part behind the 50% increase of *|Z|* in the presence of non-toxic concentrations of doxorubicin, but possibly other factors regarding drug-induced extracellular events might also contribute to such an impedance increase at LF.

In addition, it was shown that the drug-induced impedance increase is concentration-dependent (Figure 8a) and reversible (Figure 8b and 8c). This implies that the drug itself might induce a direct effect on cellular structure, and/or cells might temporarily adopt a protection mechanism against the drug effects by their structural reorganization *via* actin remodeling. Overall, potential stimulatory drug effects such as cell stiffness were measured during the continuous exposure of cells to the nontoxic drug concentrations. These findings suggest that impedance spectroscopy is suitable for detecting and quantifying the increasing cell stiffness of drug sensitive cancer cells upon drug exposure and sensing the alterations of biomechanical properties of cancer cells on the onset of drug resistance.

### Conclusions

In conclusion, we demonstrated that drug resistant breast cancer cells could be distinguished from their parental cell population based on the differences in their dielectric properties such as *R_extra_*. It was also shown that these two cell lines demonstrate a sharply different impedance response to doxorubicin. Moreover, a reversible increase in the cell stiffness in the presence of nontoxic drug concentrations could be measured using impedimetric measurements, indicating that structural reorganization of F-actin due to the drug could play a significant role on the onset of gaining drug resistance. Such stimulatory response with simultaneous LF and HF analysis enabled the creation of an impedimetric cellular drug response map. Future investigations might include testing heterogeneous tissue samples from patients and the use of multiple assays running in parallel within an automatic system in order to develop a high-throughput platform that meets the needs of laboratory diagnostics and pharmaceutical industry more comprehensively.

Overall, impedance screening is a trendsetting technique owing to a number of striking advantages including label-free, non-destructive, cost-effective and continuous cell characterization. From this point of view, cell-based impedimetric sensing opens new avenues in personalized medicine to create new classes of detection devices for drug induced cellular events. Such sensing of cellular phenomena using impedance analysis combined with real-time imaging might represent a useful technique for identification of cancer cells at different stages and their interaction with drug during the disease. Thus, such miniaturized devices could be useful for personalized medicine applications to identify the disease progress and optimize the drug treatment of a patient during chemotherapy.

## Supporting Information

Figure S1 **Electrode design; a bipolar interdigitated electrode (IDE) pair were adapted to six-filter configuration to maximize electrode surface coverage for high-sensitivity impedance measurement of the cell culture area.** Total of 54 electrode fingers are provided for each electrode (w = 10 µm, l = 100 µm) separated by a gap of 15 µm.
(TIF)

Figure S2 **Cell adherence on microelectrodes monitored as a function of time by impedance spectroscopy at 10 kHz for a) MCF-7 WT b) MCF-7 DOX.** Imaging of cell culture on microelectrodes before drug treatment for c) MCF-7 WT and d) MCF-7 DOX.
(TIF)

Figure S3 a) The impedance profile of cell medium with and without doxorubicin in the absence of cells at 10 kHz as a control experiment. 5% increase in the impedance magnitude was observed when 20 µM drug was introduced to the cell medium; b) The temporal evolution of |Z| of 20 µM doxorubicin in cell medium in the absence of cells, no change was observed in the impedance as a function of time at 10 kHz.
(TIF)

Figure S4 **Imaging of MCF-7 WT cells on microelectrodes before and after drug treatment of a) 0.2 µM doxorubicin b) 2 µM doxorubicin c) 20 µM doxorubicin.** (a) 8 h and 24 h of 0.2 µM drug treatment caused morphological changes such as cell retraction (blue arrow) but no cell death was observed (b) Cell retraction (blue arrow) and formation of wider intercellular gaps (red arrow) were observed after 8 drug treatment and some cell death occurred (black arrow) after 24 h of 2 µM drug treatment. (c) Severe morphological changes (red arrow) and cell death (black arrow) took place both after 8 h and 24 h treatment of 20 µM doxorubicin.
(TIF)

Figure S5 **Imaging of MCF-7 DOX cells on microelectrodes before and after drug treatment of a) 20 µM doxorubicin; no cell death was observed after 24 h and cells were healthy and highly densely packed on the microelectrodes.** b) No doxorubicin (control); cells were healthy and densely packed after 24 h.
(TIF)
