# Supplementary figures and images for: Label-Free Recognition of Drug Resistance via Impedimetric Screening of Breast Cancer Cells
Source: PLoS One. 2013 Mar 4;8(3):e57423. doi: 10.1371/journal.pone.0057423 (PMC3587579; doi:10.1371/journal.pone.0057423)

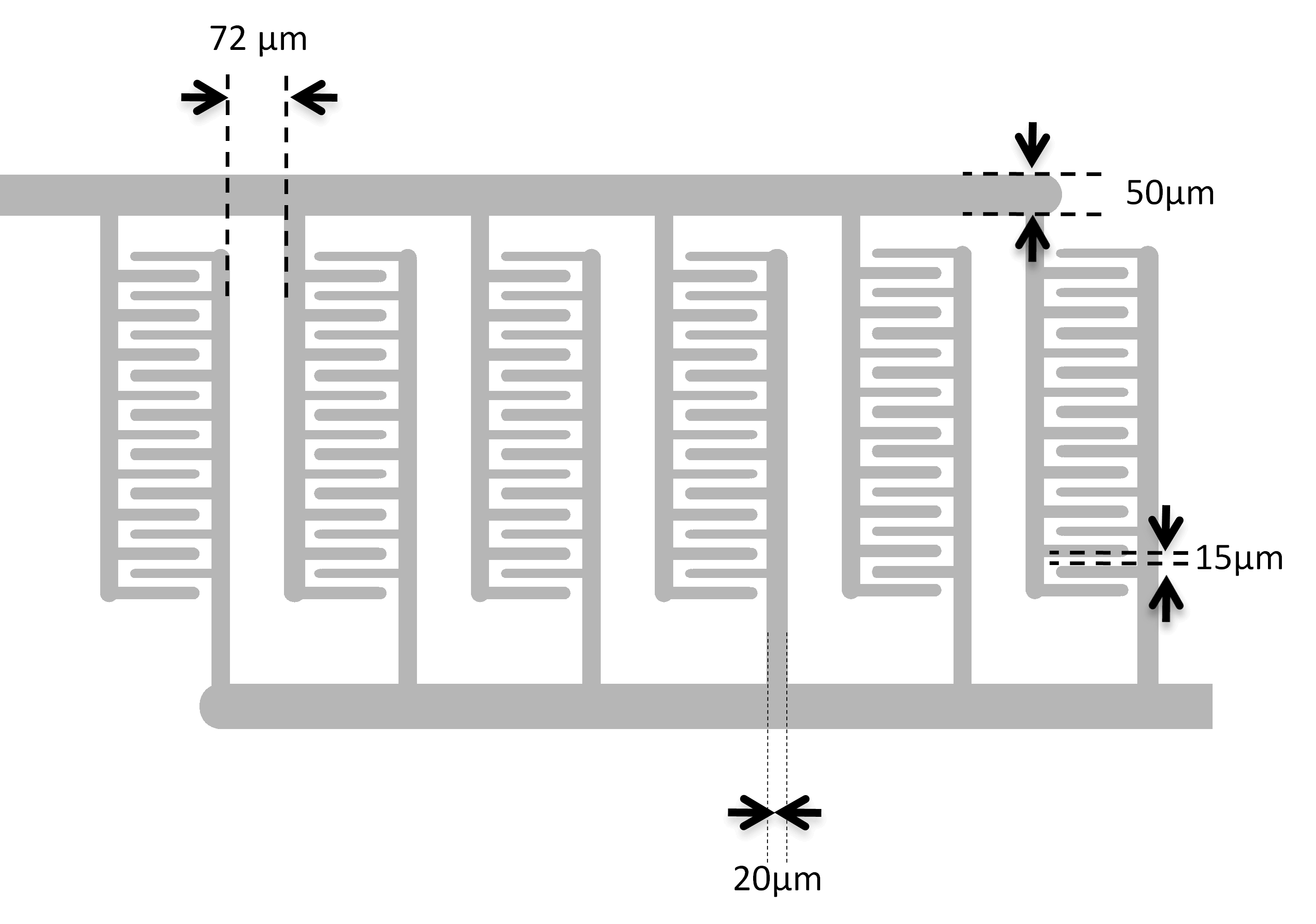

Supplement: Figure S1 — Electrode design; a bipolar interdigitated electrode (IDE) pair were adapted to six-filter configuration to maximize electrode surface coverage for high-sensitivity impedance measurement of the cell culture area. Total of 54 electrode fingers are provided for each electrode (w = 10 µm, l = 100 µm) separated by a gap of 15 µm. (TIF) [file pone.0057423.s001.tif]

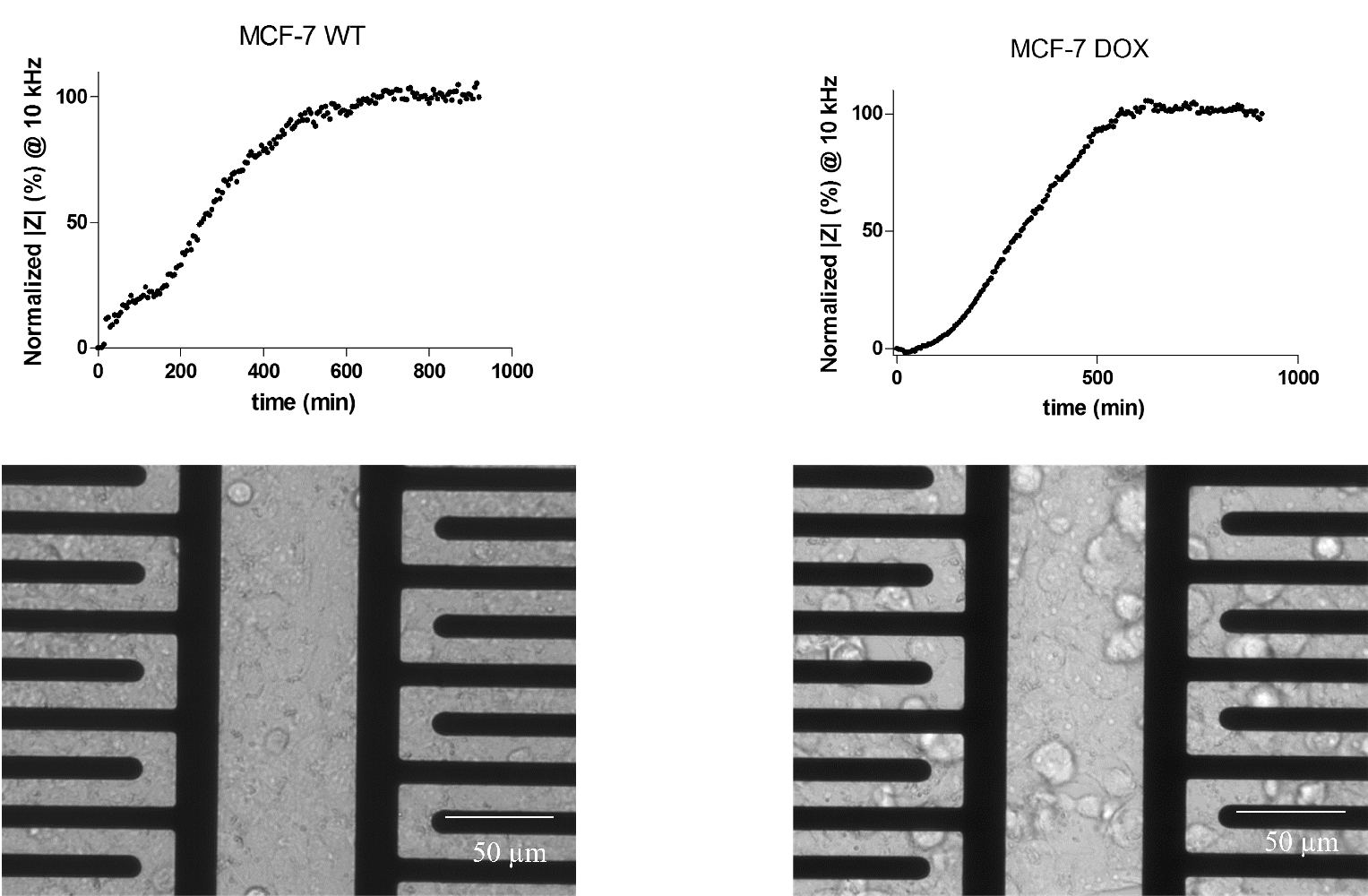

Supplement: Figure S2 — Cell adherence on microelectrodes monitored as a function of time by impedance spectroscopy at 10 kHz for a) MCF-7 WT b) MCF-7 DOX. Imaging of cell culture on microelectrodes before drug treatment for c) MCF-7 WT and d) MCF-7 DOX. (TIF) [file pone.0057423.s002.tif]

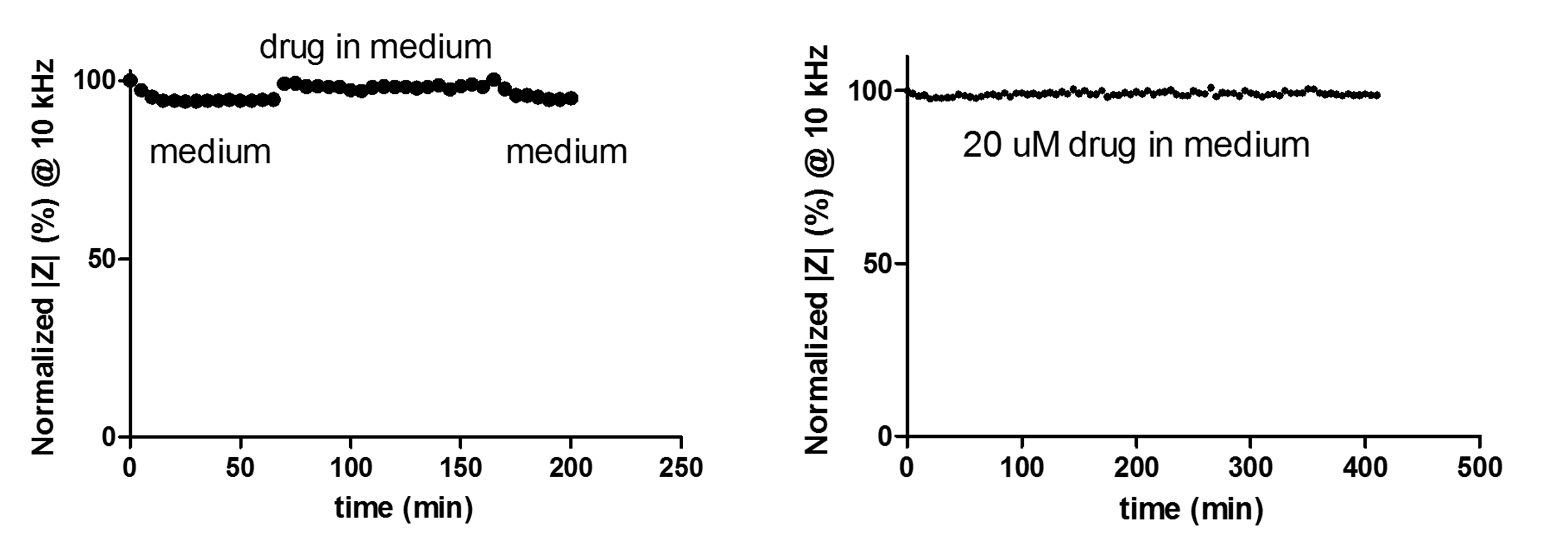

Supplement: Figure S3 — a) The impedance profile of cell medium with and without doxorubicin in the absence of cells at 10 kHz as a control experiment. 5% increase in the impedance magnitude was observed when 20 µM drug was introduced to the cell medium; b) The temporal evolution of |Z| of 20 µM doxorubicin in cell medium in the absence of cells, no change was observed in the impedance as a function of time at 10 kHz. (TIF) [file pone.0057423.s003.tif]

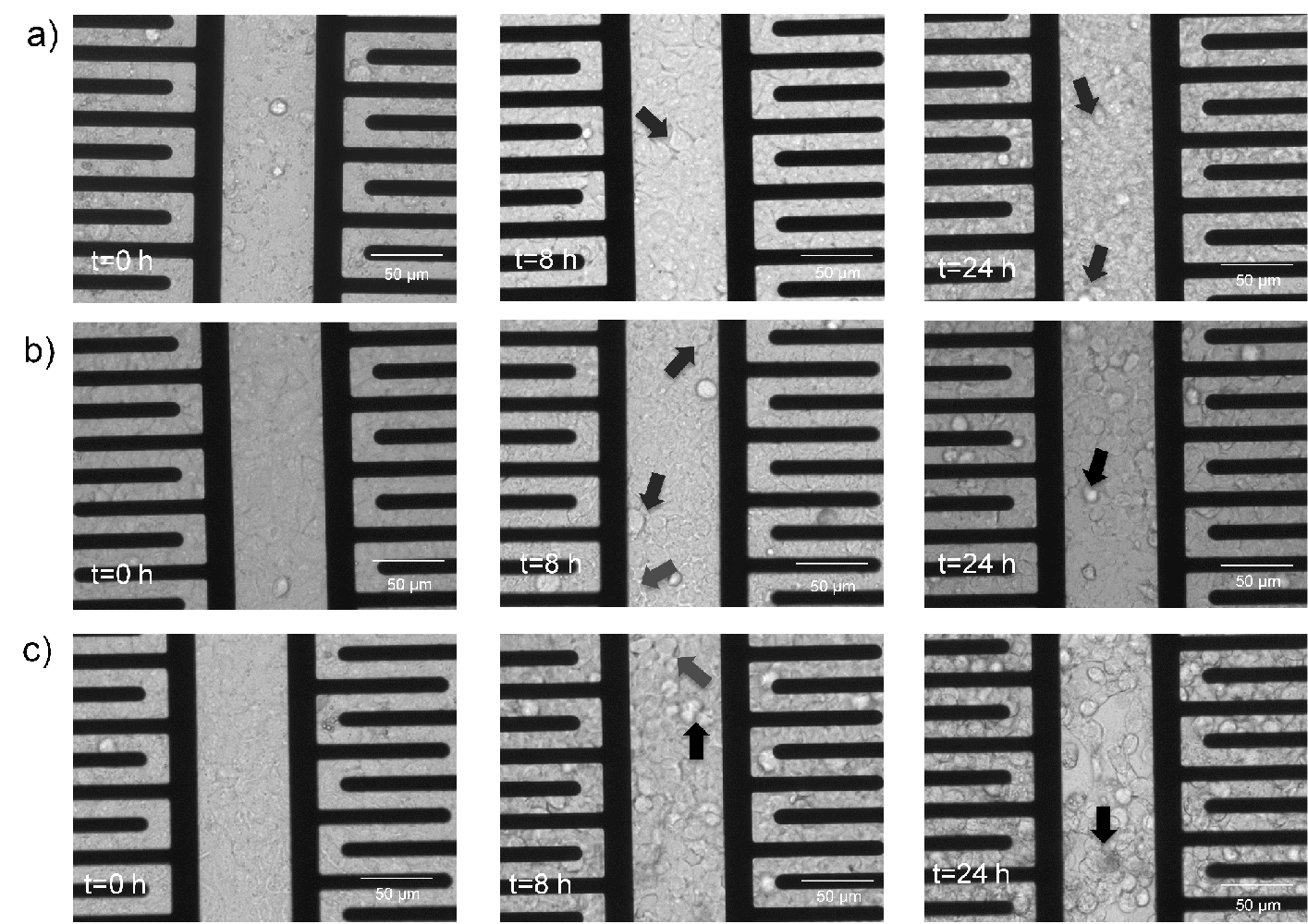

Supplement: Figure S4 — Imaging of MCF-7 WT cells on microelectrodes before and after drug treatment of a) 0.2 µM doxorubicin b) 2 µM doxorubicin c) 20 µM doxorubicin. (a) 8 h and 24 h of 0.2 µM drug treatment caused morphological changes such as cell retraction (blue arrow) but no cell death was observed (b) Cell retraction (blue arrow) and formation of wider intercellular gaps (red arrow) were observed after 8 drug treatment and some cell death occurred (black arrow) after 24 h of 2 µM drug treatment. (c) Severe morphological changes (red arrow) and cell death (black arrow) took place both after 8 h and 24 h treatment of 20 µM doxorubicin. (TIF) [file pone.0057423.s004.tif]

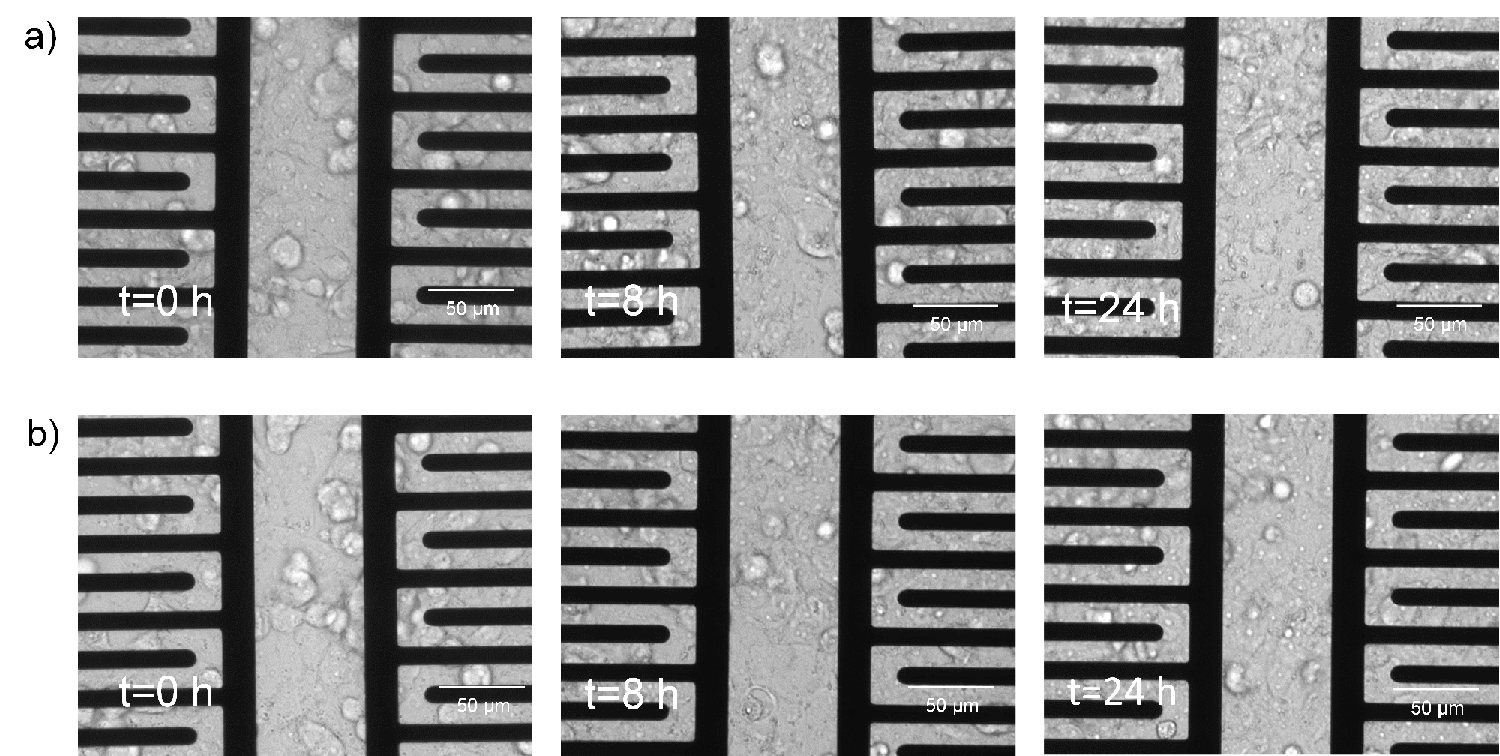

Supplement: Figure S5 — Imaging of MCF-7 DOX cells on microelectrodes before and after drug treatment of a) 20 µM doxorubicin; no cell death was observed after 24 h and cells were healthy and highly densely packed on the microelectrodes. b) No doxorubicin (control); cells were healthy and densely packed after 24 h. (TIF) [file pone.0057423.s005.tif]
